# Treatment of Headache1President’s address before the Central New York Medical Association, Syracuse N. Y., October 15, 1895.

**Published:** 1895-12

**Authors:** Floyd S. Crego

**Affiliations:** Professor of insanity and diseases of the brain and clinical instructor in nervous diseases in the Medical Department of the University of Buffalo


### BUFFALO MEDICAL JOURNAL.

VOL. XXXV. DECEMBER, 1895. No. 5.

### Original Communications.

### TREATMENT OF HEADACHE.[1]

BY FLOYD S. CREGO, M. D.,

Professor of insanity and diseases of the brain and clinical instructor in nervous diseases in the Medical Department of the University of Buffalo.

BEFORE presenting to you the subject which constitutes my address as president of this society, I desire to express to you my heartfelt thanks for the honor conferred upon me, by selecting me as your presiding officer. The work which has been accomplished by this society in the twenty-eight years of its existence, cannot be over-estimated. Many papers are presented of scientific value each year and great good has been accomplished in engendering professional and fraternal feeling by the society.

The subject to which I direct your attention is a discussion on the treatment of the severer forms of headache. Headache, as a general rule, is the result of various diseases and hence is only a symptom, and " we know almost nothing of the structures in which the pain of headache is felt, or the mechanism of its production." The subject is one on which it is easy to theorise, but there are no facts that give to any hypothesis a considerable degree of probability. One conclusion is, however, suggested by the symptoms that the seat of the pain differs in various cases. Some writers would have us believe that the dura alone is the sensitive portion of the contents of the skull, with the exception of certain cranial nerves. Again, we are told all of the membranes and the cortex may be the seat of pain. As I have already stated in the above quotation from a celebrated author, all theories as to the seat of the pain in headache is mere speculation, but many things point to the fact that any of the membranes of the cortex and the

1. President's address before the Central New York Medical Association, Syracuse N. Y., October 15, 1895.

cerebral structure itself may be the seat of the pain. "Whether pain is produced directly by morbid states of the cerebral tissue we cannot say." We are certainly not justified, however, in denying this seat of pain. It may be caused by organic processes which do not involve the surface. "After a necrotic softening in the ganglia, headache is common on the side opposite to the hemiplegia and the diffuse pain of hemicrania may be on the side opposite to that of the sensory symptoms." Many of the morbid states which give rise to headache are found in the fact that the brain is shut up in an unyielding cavity and while the weight of the brain is as but one to forty, one-fifth of all the blood in the body circulates in the brain constantly, and frequently, through vascular relaxation, the brain becomes suddenly engorged with blood, or, on the other hand, through weakening of the heart's action the cranial cavity is more liable than any other organ to be slowly filled with improperly oxygenated blood and irritation of the cortex takes place. The brain is frequently furnished with improper and deficient quality of blood, which rapidly gives rise to molecular changes and disturbances of the nerve cells. Again, the brain is irritated through various organs by which it is connected by the nerves, although this is not so common as many authors would lead us to suppose. Chief among them is the eye, the stomach and the uterus. Headache is invariably the result, too, of growths in the cranial cavity and of inflammation of any portion of its membranes or the substance of the brain. It is also produced by the action of toxicants and of drugs. A continuity of causes oftentimes produces the same results and we see this exemplified in headache as well as in many other diseases. There is first the influence of excitement and over-stimulation to the brain and then the influence of exhaustion. "When both act in concert, how rapid is the downfall."

The subject of headache is very perplexing; as we have said, it is but a symptom and we have to look for the cause in other organs, and when apparently we have discovered the cause, we attempt to remedy it by treating the seat of the disease and we often fail. This is demonstrated very clearly in headache due to eye-strain. We all know that a vast majority of headaches we are called upon to treat are due to eye-strain and yet, when we attempt to relieve the headache by the proper adjustment of eye-glasses, or cutting of the muscle, we do not accomplish our purpose, for we find through this eye-strain that other functions have been disturbed, such as digestion and functional disease of the stomach has been induced and through improper action of this organ we have malnutrition, and malnutrition gives poor blood, and poor blood gives poor blood-supply to the brain, and if this continues for a long time, molecular change and instability of the nerve cells. Thus we see in the treatment of such a case, what a vast territory we have to cover. First, of course, we have a competent oculist correct the eye-strain and then direct our attention to other functions which have been deranged. Here another difficulty meets us. Our patients become restless under prolonged treatment and go from one physician to another, giving none a proper opportunity to accomplish anything. Another difficulty we encounter in the treatment of headaches is that we have no objective symptoms, only subjective. Pain is pain to a person who suffers from headache; it has no degree by which it can be gauged, and the aching cerebral mass shut up in the bony cavity only reveals its morbid condition by the statements of the sufferer. Thus there is no disease or symptom which taxes the experience or scientific knowledge of a physician more than this and no case can be fathomed, as a general rule, without close observation and study, but it is not within the province of this paper to discuss many of these conditions, but rather to direct your attention to a few of the severer forms of headache.

Before proceeding, I wish to exhibit to you a diagram, which is very accurate, according to my experience, and which is taken from Dana's work on nervous diseases, which shows the location of the pain in headaches from different causes. Of this, Dana says: "The accompanying diagram shows some of the relations of localised pain to the cause. A large experience, both in my own practice and that of others, shows it to be approximately correct." As already stated, I consider this quite correct and of diagnostic value.

The first form of headache to which I direct your attention is megrim or migraine. This form of headache is sometimes called sick or bilious headache. The first name is given it on account of a persistent symptom of the disease and the second, bilious, points to a theory of causation which we shall see later on, carries us back to the time when the ancient and now for many years obsolete humoral pathology still prevailed. This disease is not very common; in fact, I might say it is rare. It is essentially an inherited disease; in all cases not direct, but the ancestors of nearly every sufferer from this disease are nervous subjects. The most severe case I ever had under treatment was that of a maiden lady whose mother had had megrim and yet all of her sisters had escaped, but several of her sister's children also had this disease in an aggravated form. We find it comes on between the ages of seven and fifteen and females are more often affected. That it is paroxysmal as well as periodic in character. The attacks in early life are so mild and of such short duration that their true character is overlooked by the physician and many will not agree with the statement that it occurs in such young subjects for this reason.

By the laity and many physicians the paroxysms are thought to be due to some gastric or digestive disturbance. Occasionally this is so, but usually the gastric disturbance is the result and not the cause. A patient, recently under treatment, had a paroxysm about every three weeks and believed the attack was caused by some indiscretion in eating, lived on milk and light soups for many weeks without any improvement in the disease. It is not dependent on menstruation, as we see it begins most always long before the menstrual epoch. In many cases we seem to have a multitude of exciting causes to bring on the paroxysm: the over-use of eyes when eye-strain is present, mental overwork, excitement, severe and sudden changes in the weather. "There is nothing in this to cause surprise when we reflect on the great physiological law, that in proportion as the nutritive and vegetative functions are feeble and languishing nervous phenomena are mobile, exalted and irregular,"—a law which has been admirably formulated in the simple observation of Hippocrates, "*sanginis moderator nervorum.*"

Therefore, whatever tends to lower the standard of health will produce, in some cases, an aggravation of the disease. Again, we see the paroxysm come on without any exciting cause, which gives credence to the theory of the disease which I will propose later on. Thus we see it is a permanent malady, lasting in many cases through the greater part of life, but is only manifested at more or less distant intervals in distinct attacks, or seizures of a well-defined character of a limited duration, leaving the patient, with few exceptions, in the enjoyment of his usual good health in the interval.

There are many theories as to the cause of the disease. The most popular in former times was the bilious or gastric, but these have now been discarded. Again, there are theories of vascular disturbance and these are three in number: active hyperemia, that is from some particular unknown cause; there is a determination of blood to the brain and a paroxysm follows. Again, venous congestion, or stasis, has been propounded and advocated as a cause. Again, irregularities of distribution from spasm or relaxation of the smaller capillary vessels is said to produce it. The theory of Dubois Raymond is that of vaso-motor spasm, and yet this could not explain all the symptoms, especially the pain.

Prof. Dubois Raymond maintained that in megrim a spasm takes place in the muscular coats of the vessels of the affected half of the head. Dr. Latham's theory refers to anemia of the brain and I should be inclined to this theory if we were forced to take a vascular disturbance as the basis of our speculation.

The theory advanced by Liveing is that of nerve storm, or nerve explosion. He claims there is a certain amount of pent-up nerve force in the brain constantly and, as a general rule, it is under our control to use as we desire, but from disease or irritation of the nerve cells this force is exploded at certain times, without a stimulus and produce many nervous phenomena; in one case epilepsy, and another megrim and so on. Thus this disease in its symptomatology exhibits "as a whole that kind of accession, culmination and subsidence which essentially belonged to epilepsy."

"On this theory, then, the fundamental cause of all neuroses is to be found, not in any disorder or irregularity of the circulation, but in a primary and often hereditary vice or morbid disposition of the nervous system itself. This consists in a tendency on the part of the nervous centers to irregular accumulation and discharge of nerve force, to disruptive and incoördinated action in fact."

"The concentration of this tendency in particular localities, or about particular foci will mainly determine the character of the neurosis in question." In the one case, epilepsy may be produced, in another, megrim. This theory of megrim is not sustained by Gowers, Dana and others, yet it has a considerable following.

Gowers maintains this is a disease primarily of the nerve cells of the higher brain centers. He says:

The hypothesis that the derangement is primarily of the brain, enables us better to understand the relation to the other neuroses and especially that to epilepsy, which is occasionally so distinct. In epilepsy, as we have seen, we must assume a disturbance of function in some cases so similar in character that we cannot doubt the identity of its seat, with that of migraine, but the process in the two differs in its course, associations and other features and these imply an essential difference in its minute character.

However, the theory that the discharging lesion is from the cortex of the posterior lobes of the brain, especially where ocular disturbance is present, or in that part of the sensory area which is the anatomical correlative of the sensation of pain in the head, is supported by many of the best authors. The symptoms are those that are present just before and after the attack and those which are present between the paroxysm. The symptoms are too well known to you all for me to review them here. It is only necessary for the purpose of treatment to keep in mind the general division.

The diagnosis of megrim is usually not difficult. It is, however, frequently mistaken for organic headache, or the headache of organic brain disease. In chronic organic brain disease we have no history of heredity and of paroxysms early in life. The pain of organic brain disease is usually always present in a mild degree and never entirely absent, although at times it becomes more severe and thus seems paroxysmal in character. The age of the patient, the history of the case and the presence in organic headache of some localised paralysis or tremor will assist us. The pain in organic brain disease is usually not unilateral.

Headache, due to malarial poisoning, is also often mistaken for megrim. Here we have the paroxysm coming on very frequently, and there is, usually, no previous history in malarial poisoning. Supraorbital neuralgia is apt to be confounded with megrim, but the symptoms of the two affections differ from each other in so many ways that they can hardly be regarded as belonging to the same category. Romberg thought the pain in hemicrania was due to hyperesthesia of the brain, and, consequently, he called it neuralgia cerebralis, in order to distinguish it from peripheral neuralgia, and, although this view is not accepted in its entirety, the opinion is gaining ground that the seat of pain is within the cranial cavity in hemicrania. All three divisions of the trigeminus send branches to the dura mater, and some maintain that here the seat of pain is located in megrim, instead of in the cerebral mass. It is certainly demonstrated that it is within the cranial cavity, while the pain in supraorbital neuralgia is outside the skull. In the treatment of the disease, we must keep in mind the fact that we have a nervous subject to deal with, and that tonics and nerve sedatives are indicated. Thus, a combined tonic and sedative treatment is of great value. Some form of iron in anemic subjects, and some form of the hypophosphites in those where simply a nerve tonic is desired, are indicated ; and, in both cases, always use a sedative. The best sedative is some form of bromide. It has been demonstrated by Clark and others, that the bromide acts on the cerebrum by quieting the action of the nerve cells. It renders them more stable and lessens the tendency to a nerve storm, hence, the continued use of a bromide in megrim is clearly of advantage. Ringer speaks highly of cannabis indica, and considers it one of the most valuable remedies that we possess for sick headache. He thinks it is most useful in preventing the attacks, not in arresting them when they have once begun. From a quarter to a half a grain of the extract, or ten minims of the tincture may be given. In this connection, I desire to mention a remedy which has been on the market for some time, which is of undoubtable value in this disease—namely, neurosine. It is a combination of the bromides with cannabis indica. The dose is a dram repeated frequently. In giving the bromides continuously, a small dose should be employed, and if acne occur, as it will in most cases, give a small dose of Fowler's solution, say three to five drops, twice a day, for a week at a time, to relieve this. The galvanic current is very useful in some cases, giving it continuously through the head with a large electrode on the forehead and a small one on the back of the neck. A very weak current should be used. The breaking of the current is recommended, but it is very harmful. The static current is of undoubted value ; it should be given over the head. In a case which I had under treatment last Summer, this was used, and succeeded in breaking up the attack quite often.

To break up the attacks, we possess at present very useful remedies — namely, antipyrin, antifebrin, phenacetin and exalgyn. The combination of the antifebrin with citrate of caffein and monobromide of camphor in a tablet called "migranine" is by far the best. About four grains of the antifebrin, one grain each of caffein and camphor may be given at a dose, and continued at intervals of four hours for a day in the endeavor to ward off the attack. They do little, if any, good when an attack has come on. The pain during an attack is best controlled by chloral or morphine. The danger of creating the morphine habit is so great that it almost entirely precludes its use, but occasionally it may be used. The chloral, however, can be given, and if vomiting prevents its administration by the mouth it is quite as readily absorbed by the rectum. After the attack and, indeed, between the attacks nitroglycerine is very highly commended.

In the majority of cases, and especially those in which there is conspicuous pallor in the early stage, this is a drug that has the most influence, given regularly during the interval, just as bromide is given for epilepsy, it has a striking effect in many patients, rendering attacks far slighter and less frequent, and often stopping them altogether. It should be given twice or three times a day after food; if taken when the stomach is empty, it passes rapidly into the blood and may cause brief cephalic discomfort, which, though not objectionable in itself, sometimes deters the patient from continuing the medicine. To avoid causing alarm, it is, therefore, desirable to begin with a small dose, one $\frac{1}{150}$ or even $\frac{1}{200}$ of a grain. The most convenient mode of administration is in the 1 per cent. solution of alcohol, now termed tincture trinitrin (B. P. Sup.). It can be given with tinctures or acids, but it is decomposed with alkalies. A very useful combination is with tincture of nux vomica, tincture gelsemium and dilute phosphoric acid, or with citrate of lithia and the acid syrup of lemons.

During the attack this should be always discontinued. Tincture gelsemium is another remedy which for many years has been employed by homeopathic practitioners in all head troubles. In this disease it is useful after the attack to allay the nervous irritation often seen.

In certain classes of cases, considerable doses of iodide of potassium with ergot are of great value. Time will not permit my calling attention to all the remedies which have been recommended, and it is needless for me to speak of the dietetic and hygienic treatment—they are well known to you. Among the many accessories, however, which are often neglected, are exercise and physical culture; too great importance cannot be given to these. Develop the child physically and we have one of the greatest adjuncts in the treatment and safeguards against the disease.

Another severe and distressing headache is that which occurs in the progress of syphilis. It is essentially a tertiary manifestation. It occurs so frequently just after or even with the secondary stage that its true character is often overlooked; indeed, it may often occur as a secondary manifestation, and when it so occurs it is rather to be classed as a toxemic condition, due to the peculiar condition of the blood. When it occurs, it is more often a tertiary manifestation, and is due to syphilis of the brain, either a neoplasm in the coat of the artery, or several arteries forming thrombi, or gummata, or as a syphilitic pachymeningitis.

The headache of cerebral syphilis may occur from one month to twenty years after the primary sore. Fournier claims that two-thirds of the cases of cerebral syphilis are of the thrombotic variety, and in this case the headache is due to cerebral anemia. As regards the length of time before syphilis may attack the nervous system, we find a diversity of opinion. Dayale is said to have found paralysis of the portio dura a month after the first symptoms of constitutional syphilis. In the case of M. X., reported by Ad. Schwarz, headache came on the fortieth day after the appearance of the primary sore and a hemiplegia the forty-sixth day. This citation of cases might be extended, but it is sufficient to say that nervous syphilis may occur, not very rarely, within two months after infection and many occur in six months. Headache in the various forms of cerebral syphilis is most distressing. It is usually localised, although occasionally general, and is easily diagnosticated by the presence of some other manifestation of the disease, such as temporary loss of power in an extremity. The ophthalmoscope will, usually, reveal a choked disc. There is also psychical disturbances and often partial epileptiform attacks. The attacks of headache are decidedly paroxysmal in character and are subject to acute exacerbations. They are always worse in the night time.

Heubner asserts that when this headache is localised it is generally made distinctly worse by pressure at certain points, but this is thought by others to exist only when the bone or periosteum is diseased.

The treatment—and my object in calling attention to this form of headache is the treatment—consists in the large doses of iodide and bromide potassium. The iodide should be pushed to the point of toleration, and this, in many cases, is almost without limit. Patients who suffer from cerebral syphilis take from 200 to 300 grains daily of the iodide of potassium without ill effects. The best method of administration is by commencing on twenty grains three or four times a day, largely diluted in water, and increased ten or twenty grains a day until sixty or eighty grains four times a day are taken. To control the pain, bromide of potassium, and, in some cases, chloral added, will be found to suffice. In many cases the acute paroxysm takes the form either of meningitis or else of a brain congestion. To relieve this, venesection should be employed. When the pulse is full and bounding and this is not resorted to, aconite and ergot are indicated.

Mercury, too, should be employed, and, when given, can be best used in the form of an inunction and as large doses as half a drachm of the unguentum hydrargyri being used once or twice a day until slight salivation is noticed, and then discontinued for a time. The galvanic current applied to the head, by the method already described, is of undoubted value. Stillingia and some of the compounds of the drug are of value, but should not be relied upon. The headache from eye-strain is another most troublesome form to treat. The pain is, in some cases, constant and, again, paroxysmal in character, coming on after the excessive use of the eyes. It is, however, seen very frequently in persons who do not use the eyes so excessively. The pain is characteristic. First, it is referred to the eyes and from there to the temples and to the back of the head, as shown in this diagram. After it comes on, it is described as a constant pressure-pain, referred to one or all of these portions of the head. The eye-strain is so frequently the cause of stomach disturbance that this is often mistaken for the whole cause of the trouble, while, in fact, it is but secondary to the eye trouble, and the digestive derangement is treated for months without any result. Again, the stomach trouble gives rise to malnutrition and cerebral anemia, and thus the physician is apt to consider it simply a condition of cerebral anemia, or irritation, due to an improper blood state. The treatment of this form of headache consists in the proper adjustment of eye-glasses to correct any focal lesion or want of proper muscular balance. The muscular strain is, in a vast majority of cases, dependent on the focal lesion, and corrects itself after the focal lesion is relieved, or it is dependent on general weakness, just as any muscle may be weak if there is any general physical derangement and weakness. To correct muscular strain, many plans have been suggested. First, tenotomy of the weaker muscles and shortening of the weaker muscle ; and the plan proposed by Ranney and Stevens, which is that of establishing muscular balance by graduated tenotomy of the stronger muscle, so many striæ of the muscle are cut at different times until the muscular strain is overcome. This plan has not been accepted by oculists in general. It has the disadvantage of making two weak muscles in place of one ; it is on the same plan as treating a case of hemiplegia or facial paralysis, due to a cerebral lesion, by producing artificially a lesion on the opposite side to give the same amount of paralysis as in the part already affected on the opposite side.

The plan adopted by oculists to overcome muscular strain is, first, correct the focal lesion; second, constitutional treatment to strengthen the muscle, and third, exercising the muscle by the use of prism to strengthen it and establish the normal power. The difficulty encountered in the treatment of these cases is in getting our patient to consult an oculist. When this is suggested, they say they were never conscious of any eye trouble; on the contrary, their eyes are remarkably good. Once they have consulted an oculist, they expect immediate relief. This is not always so readily obtained, and patients not receiving the relief they imagined they should, believe that eye-strain is a myth. In some cases the relief is immediate after the proper adjustment of the eye-glass, but in the vast majority of cases other symptoms which have resulted from the eye lesion must first be corrected to give the desired result. The treatment of these conditions is made very easy with the aid afforded by the oculist, and stomach derangements and systemic disturbances disappear under appropriate treatment where before they were intractable.

469 Delaware Avenue.

